# A Memory of Majorana Modes through Quantum Quench

**DOI:** 10.1038/srep29172

**Published:** 2016-07-08

**Authors:** Ming-Chiang Chung, Yi-Hao Jhu, Pochung Chen, Chung-Yu Mou, Xin Wan

**Affiliations:** 1 Physics Department, National Chung-Hsing University, Taichung, 40227, Taiwan; 2 Physics Division, National Center for Theoretical Science, Hsinchu, 30013, Taiwan; 3 Physics Department, National Tsing Hua University, Hsinchu, 30013, Taiwan; 4 Institute of Physics, Academia Sinica, Taipei 11529, Taiwan; 5 Zhejiang Institute of Modern Physics, Zhejiang University, Hangzhou, 310027, P. R. China

## Abstract

We study the sudden quench of a one-dimensional p-wave superconductor through its topological signature in the entanglement spectrum. We show that the long-time evolution of the system and its topological characterization depend on a pseudomagnetic field **R**_eff_(*k*). Furthermore, **R**_eff_(*k*) connects both the initial and the final Hamiltonians, hence exhibiting a memory effect. In particular, we explore the robustness of the Majorana zero-mode and identify the parameter space in which the Majorana zero-mode can revive in the infinite-time limit.

The implementation of a quantum computer requires fault-tolerant quantum information processing. Conventional quantum error correction codes^1^ demand an error threshold that is still beyond the reach of the current technology. Alternatively, one may exploit exotic topological excitations that obey non-Abelian braiding statistics to encode quantum information, which would then be robust against local perturbations. Named after Ettore Majorana^2^, Majorana zero-modes seem to be the easiest to construct among the family of objects that realize non-Abelian statistics. A prototypical model for Majorana zero-modes is the one-dimenaional (1D) p-wave superconductor^3^, in which different topological phases can be characterized by a topological *Z*_2_ index. Topological non-trivial phases can be identified by the presence of zero-energy Majorana edge modes at open boundaries, which may be realized at the interface of superconductors with either topological insulators^4^,^5^ or semiconductors with strong spin-orbit coupling^6^,^7^,^8^. Recent experimental progress^9^ further fuels the interest in the preparation and manipulation of Majorana zero-modes.

To control the Majorana zero-modes for braiding or computing one needs to dynamically change the experimental parameters, such as the gate voltage in a wire network^10^ or the magnetic flux in a hybrid Majorana-transmon device^11^. This motivated the study of the out-of-equilibrium dynamics of systems with Majorana modes at the ends^12^,^13^,^14^,^15^, which found that topology could induce anomalous defect production that could cause quantum decoherence, when a system was adiabatically driven through a quantum critical point. On the other hand, a sudden quench (not necessarily on a topological system) gives rise to the question whether the system can undergo relaxation to an equilibrium state upon a change of parameters. Interestingly, recent studies also showed that the quench dynamics of integrable systems has a memory effect: it reaches a steady state depending strongly on the initial condition^16^,^17^,^18^,^19^. However, the majority of the studies along that line mostly concern the bulk properties while only few studies also investigate the edge properties^20^,^21^,^22^. In order to use Majorana zero-modes as robust quantum information carriers, it is, hence, of great interest to study the stability of the Majorana edge modes after sudden quenches.

An alternative avenue that connects Majorana zero-modes and quantum information is quantum entanglement. The common entanglement measurement is the von Neumann entropy of a subsystem *A*: *S*_*A*_ = −Tr *ρ*_*A*_ log_2_ *ρ*_*A*_, where 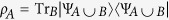 is the reduced density matrix after tracing out the environment *B* from the whole system *A* ∪ *B*. For a topological system the size-independent constant of the entanglement entropy is related to the total quantum dimension^23^,^24^, which can be used to detect topological order. Nevertheless, more information is revealed in the entanglement spectrum, i.e. the eigenvalues of the entanglement Hamiltonian 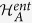, whose thermodynamic entropy at “temperature” *T* = 1 is equivalent to the entanglement entropy^25^. Under certain circumstances one can view, at least on the low-energy scale, the entanglement Hamiltonian on the subsystem *A* with the open boundaries as the deformed real-space Hamiltonian that preserves the topological information, hence the presence of zero-energy Majorana edge modes can be detected by a corresponding degeneracy in the entanglement spectrum; more precisely, a pair of doubly degenerate eigenvalues of 1/2 in the one-particle entanglement spectrum^26^,^27^,^28^,^29^. This provides a reliable measurement of the Majorana edge modes.

In this report we fuse two subjects by exploring the quench dynamics of Majorana zero-modes of a 1D p-wave superconductor under the entanglement measurement process, thereby offer a quantum information perspective of the manipulation of topological systems and the robustness of the Majorana zero-modes under sudden quench. We find that the topology of the infinite-time behavior can be determined by the properties of a pseudomagnetic field **R**_eff_, which connects both the initial and the final Hamiltonians, hence exhibiting a memory effect. In general, a quench across any phase boundary will not give rise to Majorana zero-modes. Surprisingly, the quench within the same topologically nontrivial phase may also lead to the loss of the Majorana modes. We provide an equation describing a critical surface in the parameter space of 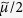, 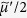, and 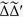. For quenches whose parameters lie above the critical surface, the initial Majorana zero-modes can revive in the long-time limit.

## Results

The 1D p-wave superconducting system of spinless fermions proposed by Kitaev^3^ is described by the Hamiltonian


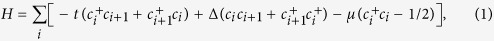


with the nearest-neighbor hopping amplitude *t*, superconducting pairing Δ, and on-site chemical potential *μ*. The translational invariant Hamiltonian (1) can be written as


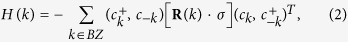


where *σ* = (*α*_*x*_, *σ*_*y*_, *σ*_*z*_) are Pauli matrices, and **R**(*k*) = (0, −Δ sin *k*, *t* cos *k* + *μ*/2) is the pseudomagnetic field. The one-particle energy spectrum is simply 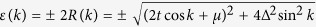. The spinless p-wave superconductor (1) breaks the time-reversal symmetry but preserves the particle-hole symmetry therefore it belongs to the class D according to the classification of topological insulators and superconductors; it can be characterized by a *Z*_2_ topological invariant^30^,^31^.

**Figure 1:**
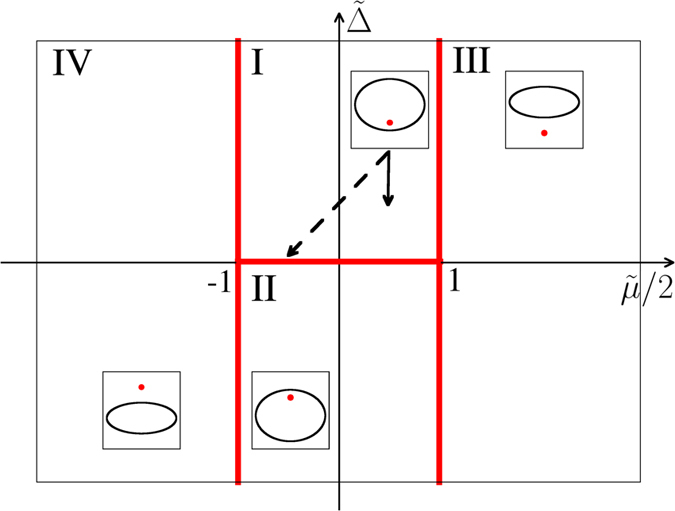
Topological phase diagram of the *p*-wave superconductor. The coordinates are defined as 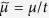 and 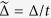. Solid arrow: quench process from (0.5, 2) to (0.5, 1) [to be discussed in Figs 4(a) and 5(b)]. Dashed arrow: quench process from (0.5, 2) to (−0.5, 0.1) [to be discussed in Figs 4(b) and 5(c)]. Insets: Representative traces of **R**(*k*) in the *R*_*y*_-*R*_*z*_ plane. In the topological phases I and II, **R**(*k*) encircles the origin (red dots), while in the trivial phases III and IV, **R**(*k*) does not encircle the origin.

The topological characterization has a simple graphical interpretation^26^. If the closed loop 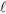 of **R**(*k*) in the *R*_*y*_-*R*_*z*_ plane encircles the origin, zero-energy edge states exist and the system is in the nontrivial phase; otherwise, the loop can be continuously deformed to a point with the bulk gap perserved, hence the system is trivial. We plot the phase diagram of the *p*-wave superconductor in Fig. 1 using dimensionless constants 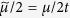 and 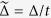. For 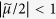, there are two different topological nontrivial phases I and II, corresponding to counterclockwise and clockwise windings of **R**(*k*) around the origin. Since the winding numbers of **R**(*k*) in phase I and II differ by two, these two phases cannot be continuously deformed to one another without closing the bulk gap, so they belong to different phases. Nevertheless, Majorana zero-modes exist at open ends in both phases. On the other hand, the states with 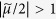 are topologically trivial and no Majorana zero-modes exist in phases III and IV.

**Figure 2:**
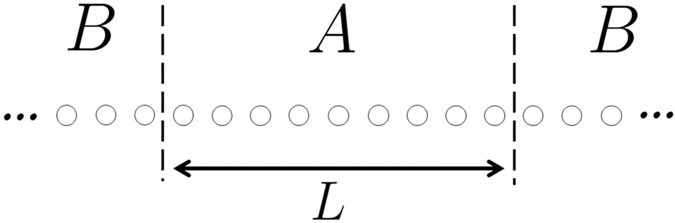
The total infinite system *AB* is divided into finite subsystem *A* with *L* sites and infinite environment *B*.

The reduced density matrix *ρ*_*A*_ can be calculated by the block correlation function matrix (CFM)^32^,^33^,^34^,^35^,^36^: 
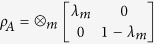
, where *λ*_*m*_ are the eigenvalues of the block correlation function matrix 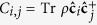 with 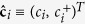 and *i*, *j* being sites of the finite subsystem *A* as shown in Fig. 2. *λ*_*m*_ is known as the one-particle entanglement spectum (OPES). For total system *AB* the bulk correlation function matrix is a 2 × 2 matrix in the Fourier space^27^. Specifically for the Hamiltonian (2) one has


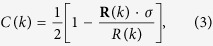


where *k* ∈ (−*π*, π]. To connect the CFM and the edge state recall that in one dimension the nontrivial Berry phase for the periodic systems in their thermodynamic limit confirms the topological nature of the systems. According to the bulk-edge correspondence^37^, two edge states with zero energy appear for the far-apart open boundaries. The same story can also happen with the entanglement spectra. The entanglement Hamiltonian *H*^*ent*^ defined as 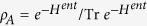 is related to the block correlation function matrix *C* in the following way^34^,^35^:


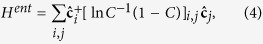


where *i*, *j* ∈ *A*. Therefore the entanglement Hamiltonian shares the same eigenvalues of the block correlation function matrix *C*. From the discussion above we know that the Berry phase of *C* in its thermodynamic limit is also determined by the pseud-magnetic field *R*. According the bulk-edge correspondence we conclude that there appear two edge states for the block correlation function matrix *C* due to the natural open boundaries of the block and therefore the entanglement spectra share the same edge states.

On the other hand, by comparing *H*(*k*) (Eq. 2) and *C*(*k*) (Eq. 3) it is evident that they have the same topological characterization. By applying the bulk-edge correspondence to both the original and entanglement Hamiltonian one see that the zero energy edge modes of the original Hamiltonian correspond to the *λ*_*m*_ = 1/2 eigenvalues of the correlation function matrix. Therefore, in the topological phases I and II, the signature of Majorana zero-modes is the degenerate eigenvalues *λ*_*m*_ = 1/2 in the OPES.

The Majorana zero-modes play an important role in the entanglement between the subsystem *A* with its environment *B*. We can calculate the entanglement entropy *E*_*S*_ for the partition as 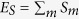 where *S*_*m*_ = −*λ*_*m*_ log_2_ *λ*_*m*_ − (1 − *λ*_*m*_) log_2_(1 − *λ*_*m*_). The pair of Majorana modes with *λ*_*m*_ = 1/2 contribute the maximal entanglement *S*_*m*_ = 1. Hence they are known as the topological maximally-entangled states (tMES)^28^,^29^.

Different from non-integrable models, which will be thermalized at infinite time, the quench dynamics of integrable models has become an important topic since such a bulk system will not be thermalized but reach a steady state decided by the initial condition described by general Gibbs ensemble (GGE). On the other hand, the Majorana edge modes are tMES, robust against perturbations, it is interesting to question how a sudden quench affect the Majorana zero-modes. Naively, if we quench from a topological phase to a trivial one, the Majorana modes may evolve into the bulk, mix with bulk modes, and disappear eventually. What happens, then, if we quench from a trivial phase to a topological one, or from a topological phase to a different one? Will the static information in the final Hamiltonian dictate the dynamical state in the long-time limit?

To answer these questions we perform a sudden quench to the p-wave superconductor at *t* = 0 by switching Δ and *μ* at *t* < 0 to Δ′ and *μ*′ at *t* > 0. This changes the Hamiltonian from *H* to *H*′ and, correspondingly, **R** to **R**′. We then calculate the time-dependent OPES *λ*_*m*_(*t*) by diagonalizing the time-dependent CFM 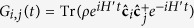 for *i*, *j* ∈ *A*. We first consider the quench processes across phase boundaries and focus on *λ*_*m*_ close to 1/2 as we are primarily interested in the fate of the Majorana zero-modes.

**Figure 3:**
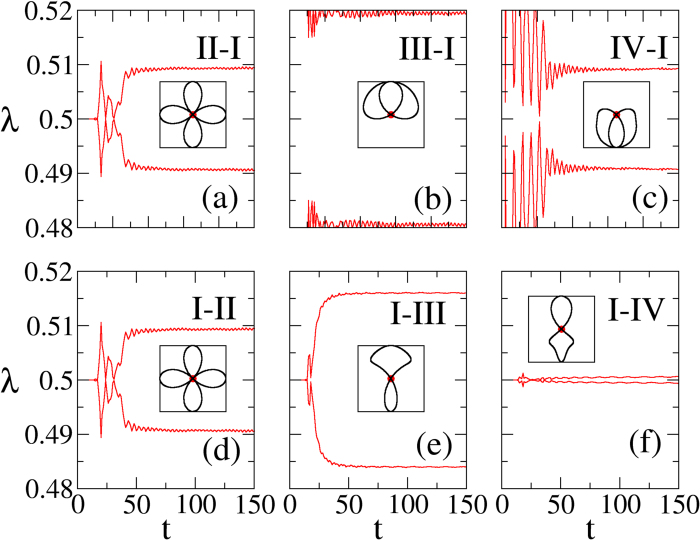
Representative time evolutions of the OPES close to 1/2 for quenches between (**a**) II-I, (**b**) III-I, (**c**) IV-I, (**d**) I-II, (**e**) I-III, and (**f**) I-IV. The initial or final parameters (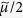, 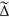) are (0.5, 2) for phase I, (0.5, −2) for phase II, (2, 2) for phase III, and (−2, 2) for phase IV. The size of the subsystem A is *L* = 100. Insets: The corresponding curves of **R**_eff_(*k*) in the *R*_*y*_-*R*_*z*_ plane. The absence of the tMES in the long-time limit is reflected by the passing of **R**_eff_(*k*) at the origin.

Figure 3(a–c) show the time evolution of the OPES near 1/2 by suddenly quenching the systems from phases II, III, and IV, respectively, to phase I. We find that the Majorana zero-modes fail to appear after a sufficiently long time, regardless of the topological properties of the original state. In other words, the quench of the topological systems with the Majorana edge modes cannot be thermalized. For comparison, Fig. 3(d–f) show the time evolution of the OPES for the sudden quench from phase I to phases II, III, and IV, respectively. The degenerate eigenvalues *λ*_*m*_ = 1/2 persist for some time before they split and relax to separate values, depending on the final Hamiltonian. In other words, the Majorana zero-modes before a sudden quench are destroyed eventually.

To further confirm the topology of the steady states of the quench process in the infinite-time limit, we analyse the time-dependent CFM in Fourier space *G*(*k*, *t*) as illustrated in the method section: Time-dependent correlation matrix. We find it can be described by a pseudomagnetic field **R**(*k*, *t*) (15) through the relation *G*(*k*, *t*) = [1 − **R**(*k*, *t*) · *σ*]/2. In the infinite-time limit the sinusoidal time dependence dephases away and *G*(*k*, *t* = ∞) depends only on the effective pseudomagnetic field


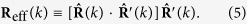


Therefore, the topology of the steady state is determined by both the initial and final Hamiltonians: the quench dynamics has a memory of the initial Hamiltonian, albeit entangled with the final Hamiltonian. The insets in Fig. 3 dipict **R**_eff_(*k*) in the above cases. We confirm that all the traces pass through the origin, indicating that the Majorana zero-modes are not stable in the infinite-time limit.

**Figure 4:**
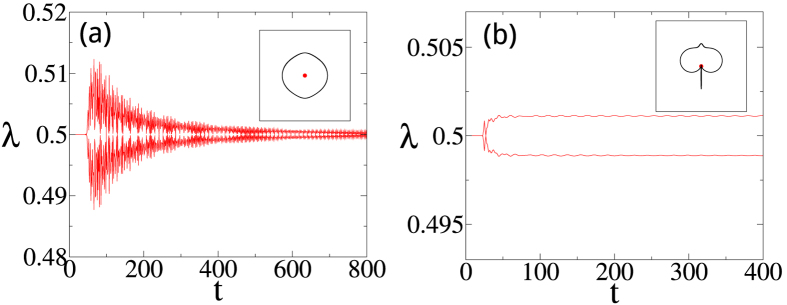
The time evolution of the OPES near 1/2 for two sudden quenches [marked by (**a**) solid arrow ((0.5, 2) to (0.5, 1)) and (**b**) dashed arrow ((0.5, 2) to (−0.5, 0.1)) in Fig. 1] within phase I. The tMES are recovered eventually in (**a**), but not in (**b**). Insets in (**a**,**b**): Traces of **R**_eff_(*k*) in the *R*_*y*_-*R*_*z*_ plane. The size of subsystem A is *L* = 100.

Will Majorana zero-modes persist if we quench between two Hamiltonians in the same topological phase? Given the fact that the edge states cannot thermalize, the naive answer of yes needs to be examined. In Fig. 4(a,b) we show the OPES evolution near 1/2 for two quantum quenches both within phase I, as indicated in the phase diagram in Fig. 1. Surprisingly, we find that the Majorana zero-modes reappear in the steady state in the infinite-time limit in Fig. 4(a), while disappear in Fig. 4(b). This contrasts to the persistence of the edge modes in a dimerized chain, where the edge mode is an electron instead of Majorana zero-modes^29^. We also plot the corresponding **R**_eff_(*k*) in the insets of Fig. 4(a,b). **R**_eff_(*k*) encircles the origin in the former case, which confirms the persistent memory of the Majorana modes after a sudden quench. In sharp contrast, **R**_eff_(*k*) passes through the origin in the latter case, which is consistent with the loss of the memory of the Majorana modes. Note that the quench processes within the phase II shall behave similarly.

**Figure 5:**
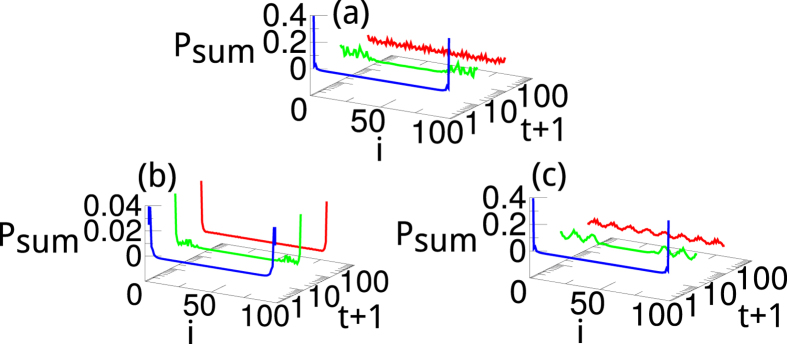
The probability sum 
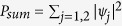
 of the two eigenstates of the entanglement Hamiltonian whose eigenvalues are closest to 1/2 at *t* = 0, 9, and 99 after a sudden quench (**a**) from I (0.5, 2) to II (0.5, −2), (**b**) from I (0.5, 2) to I (0.5, 1) as in Fig. 4(a), and (**c**) from I (0.5, 2) to I (−0.5, 0.1) as in Fig. 4(b).

To support our findings, we further analyze the eigenstates of the after a sudden quench. In Fig. 5 we plot the probability sum 
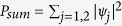
 of the two states *ψ*_1,2_ whose eigenvalues are closest to 1/2 at *t* = 0, 9, and 99. As expected, *P*_*sum*_ exhibits sharp peaks due to the presence of the Majorana edge modes. The only case that such peaks survive [Fig. 5(b)] is the quench process within phase I as discussed in Fig. 4(a). For comparison, such peaks dissolve into the bulk when we quenches the system to a different topological phase [Fig. 5(a)] or within phase I but with mismatching superconducting gaps [Fig. 5(c)].

## Discussion

**Figure 6:**
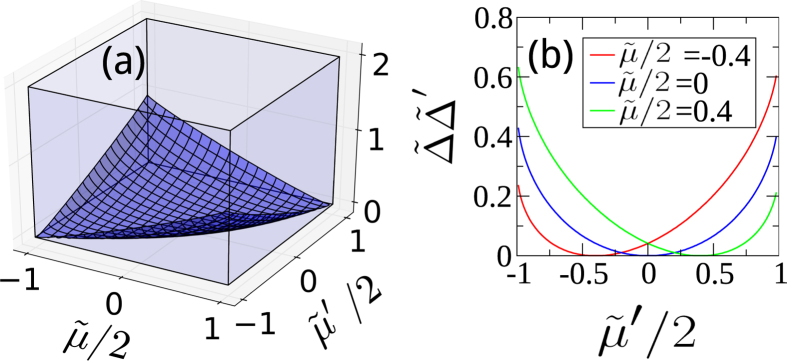
(**a**) The critical surface in the space of 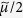, 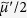, and 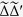 above which **R**_eff_(*k*) = 0 has no solution. (**b**) Cross sections of the critical surface at 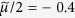, 0, and 0.4.

We have shown in the result section that the maximally entangled states (or Majarona zero modes) can still disappear at *t* → ∞ even for the quench between the same topological phase. As hinted from (5), this is due to the condition 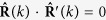 happens at some *k*. Explicitly, this means


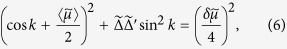


where  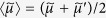 and 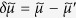. Figure 6(a) shows the critical surface below which the equality can be satisfied for some *k*; while above the surface **R**_eff_(*k*) encircles the origin (to ensure that both the initial and final Hamiltonians are in the topological phase, we also need 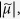

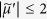
), hence the Majorana zero-modes are memorized in the long-time limit. To understand why 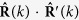 can vanish at some *k*, we find it is instructive to consider the velocity of the **R**-vectors. Intuitively, if the velocity profiles of two **R**-vectors are similar then it is impossible to have 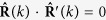. In contrast, if the maximal angular velocity of **R**(*k*) and **R**′(*k*) occur at different *k* points then it is possible to have 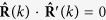. For example, if **R**(*k*) rotates rapidly while **R**′(*k*) rotates slowly then they should become perpendicular at some *k*. As shown in the method section: Maximal angular velocity and band gap, we find the position of the band gap and the maximal of the angular speed *ω*(*k*) = *dR*(*k*)/*dk* are at the same *k* (can be slightly different for some special parameter region). Since the position of the band gap can be tuned by the chemical potential *μ*, one can make the maximal angular velocity of **R**(*k*) and **R**′(*k*) occur at different *k* by tuning the chemical potential of the initial and final Hamiltonian. Thus, for the quench within the same topologically non-trivial phases (both **R** and **R**′ vectors rotate clockwise or counter-clockwise), it is still possible to make **R** rotates slowly (rapidly) while **R**′ rotates rapidly (slowly) at a particular *k*, leading to 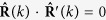. A simple physical picture to understand this phenomenon is as follows: if the superconducting gaps of the initial and final Hamiltonians have no energy overlap, the Majorana modes are suppressed due to the mismatch of the corresponding single-particle states.

For the early time behavior, one may ask what determines the finite survival time of the degenerate *λ*_*m*_ = 1/2 in Fig. 3(d–f). We can imagine that upon quench quasiparticles are generate in the bulk and propagate at a maximum velocity *v*_max_ = [∂*ε*(*k*)/∂*k*]_max_. Therefore, at 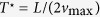 the Majorana zero-modes at the boundaries of the entanglement cut can exchange information and hence the degenerate levels in the OPES start to split. Chaotic oscillations then emerge in the entanglement spectrum due to the complex processes of quasiparticle interference and decoherence.

**Figure 7:**
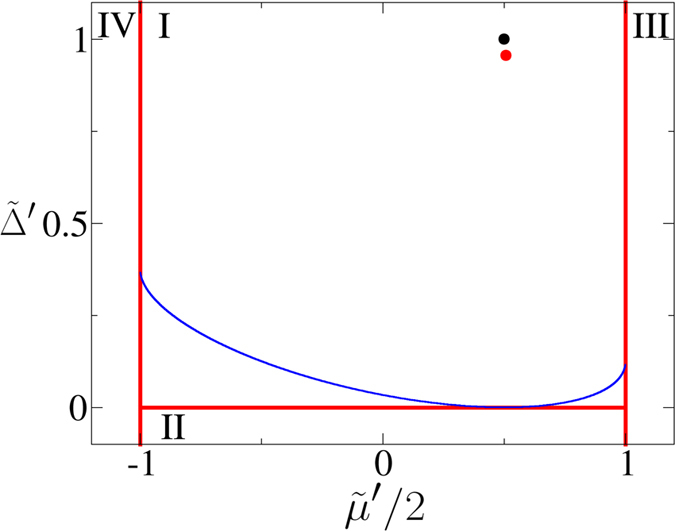
The cross section of the critical surface shown in the phase diagram of 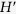 with fixed 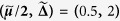. The blue curve distiguished two different steady states with (above) or without (below) edge states. Using the black point as referenced parameter, the renormalized parameters 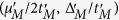 using *V* = 0.1 are shown as the red point.

**Figure 8:**
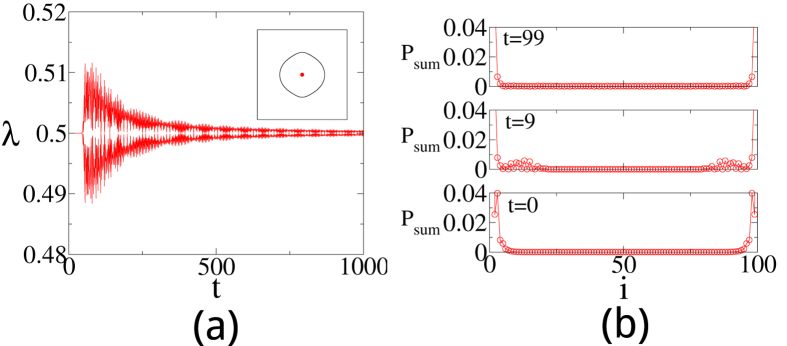
(**a**) The two OPES as function of time *t* after a sudden quench that are closest to 1/2 for the perturbation and the inset shows the effective *R* vector in the *R* space. (**b**) The probability sum 
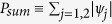
 at time *t* = 0, 9 and 99 for the perturbation.

Finally, we discuss the robustness of our result under the perturbative Coulomb interactions: 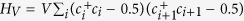 for the final Hamiltonian. Within the mean-field approach, the effects of the interaction is to renormalize the hopping, paring and chemical potential, resulting an effective mean-field Hamiltonian of the form of the un-perturbed Hamiltonian. As a result, the renormalized hopping 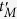, paring 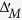 and chemical potential 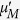 of 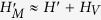 are given by the self-consistent equations (7)


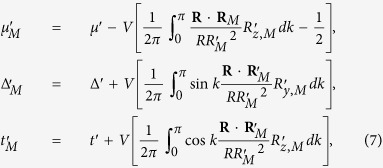


where 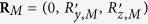 are the pseudomagnetic fields of 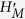 with the form given by (2). To understand the effect of the mean-field approach, the same example as Fig. 4(a) (same as Fig. 5(b)) is considered in the follow calculation. Considering *V* = 0.1 for the final Hamiltonian, we obtain 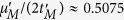 and 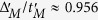 corresponding to the movement from the initial black point to the red one in Fig. 7. However this change under perturbation does not cross the critical surface which is plotted as the blue curve in Fig. 7. The results are confirmed by Fig. 8, where the two OPES *ψ*_1_ and *ψ*_2_ near 1/2 and the probability sum 
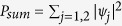
 at *t* = 0, 9, and 99 for the red point are shown. As far as the final Hamiltonian is far away from the critical surface, the perturbations of the interaction do not affect the revival of the tMES. The same considerations also apply for the perturbation of the pairing and chemical potential.

In summary, we study the quench dynamics of a 1D p-wave superconductor using the OPES. We find that the system reach a final steady state whose topology can be determined by an effective pseudomagnetic field **R**_eff_(*k*) (5). As expected, sudden quenches from a topological phase to a trivial phase destroy the Majorana edge modes. However, the memory of the Majorana modes will also be lost if we quench the system to a different topological phase, or if the superconducting gaps before and after a sudden quench do not match. When both topological and energetic criteria are satisfied, the Majorana zero-modes will return after sufficiently long time.

## Methods

### Time-dependent correlation function

To obtain the time-dependent correlation function matrix 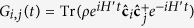, one has to diagonalize *H* (2) using


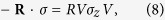


where


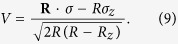


Therefore, the diagonal basis 
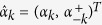
 is defined as


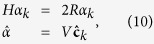


where 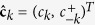. Assume the notation with and without ′ using the parameter set (*t*, *μ*, Δ) before and (*t*′, *μ*′, Δ′) after a sudden quench respectively, the time-dependent CFM after a sudden quench in the Fourier space


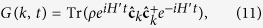


can be calculated by canonically transforming 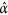 twice to the proper operators using (9) and obtain


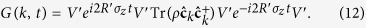


For zero temperature, *ρ* is the density matrix of the ground state, therefore


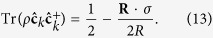


Substituting (13) and (9) into (12) and using the properties of Pauli matrices (**R** · *σ*) (**R**′ · *σ*) = **R** · **R**′ + *i*(**R** × **R**′) · *σ*, we end up with a simple form


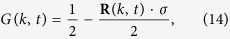


where the effective pseudomagnetic field


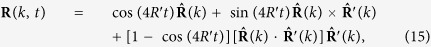


where 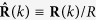 and 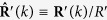.

### Maximal angular velocity and band gap

**Figure 9:**
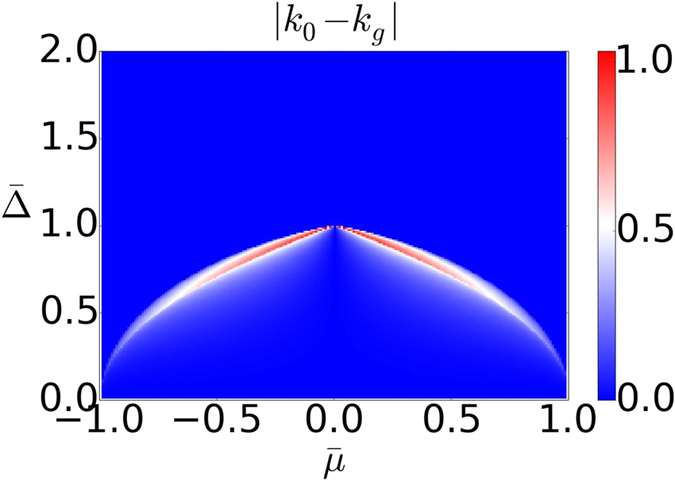
Plot for |*k*_0_ − *k*_*g*_|. In most of the parameter regime, they are the same.

Here we are going to determine the momentum *k*_0_ at which *ω*(*k*) reaches global maxima and the momentum *k*_*g*_ at which the band gap of the dispersion *ε*(*k*) is located. Then, we illustrate an interesting relation between angular velocity *ω*(*k*) and the dispersion *ε*(*k*): that one has *k*_0_ = *k*_*g*_ in some parameter regimes and *k*_0_ ≈ *k*_*g*_ in other parameter regimes as shown in Fig. 9. This provides an intuitive picture on why the topology of the steady state is determined by the initial and final Hamiltonian as discussed in the discussion section.

In the following, we will use the dimensionless parameters 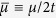, 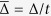 and set *t* = 1 as our unit of energy. Furthermore, we will assume that 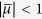 since we only want to consider the region of topologically non-trivial phases.

#### Extrema of the angular velocity *ω*(*k*)

To identify the momentum *k*_0_ at which the angular velocity *ω*(*k*) reaches its maximum, we start from the pseudomagnetic field


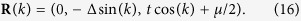


In terms of the dimensionless constants defined above it can be rewrite as


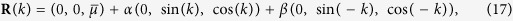


where 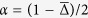, and 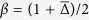. This shows that **R**(*k*) can be decomposed into as constant vector in the *z*-direction, and rotating and counter-rotating vectors in the *y*-*z*-plane. Let *θ*(*k*) be the angle between **R**(*k*) and the *z*-axis, then one has


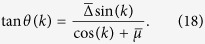


Define the angular velocity as *ω*(*k*) = *dθ*(*k*)/*dk*. By differentiating the equation above we obtain the expression of angular velocity


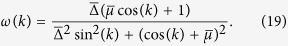


The *k*_0_ at which the angular velocity *ω*(*k*) reaches its maximum should satisfy 
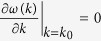
. It is straightforward to show that


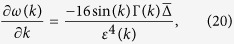


where


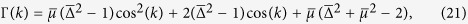


and *ε*(*k*) is the dispersion. We note that due to the finite band gap, the denominator is always non-zero. From Eq. (20) we find *k*_0_ = 0 or *k* = *π* or


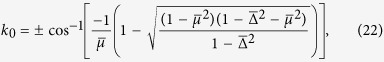


provided that


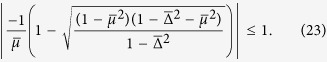


Note that in Eq. (22) we have discarded the solution with positive sign in front of the square root because it does not lead to a valid solution.

#### Extrema of the dispersion *ε*(*k*)

To identify the momentum *k*_*g*_ at which the band gap is located we start from the dispersion


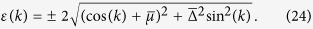


The *k*_*g*_ should satisfy 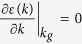, where


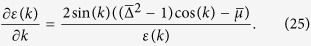


This leads to


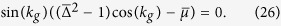


Solving for *k*_*g*_ we find *k*_*g*_ = 0 or *k*_*g*_ = *π* or


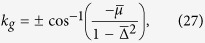


provided that


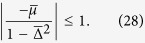


#### Relationship between *k*_0_ and *k*_*g*_

With the solution of *k*_0_, the location of the maximal angular velocity *ω*(*k*) and *k*_*g*_ the location of the gap of the dispersion *ε*(*k*) we are now is position to illustrate the close relation between *k*_0_ and *k*_*g*_. In Fig. 9 we show the difference |*k*_0_ − *k*_*g*_| as a function of 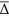 and 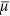. We find that *k*_0_ and *k*_*g*_ are almost the same in most of the parameter regime. In the following we provide more insight on why this happens via analyzing the solutions in various limits.

• Case 1: *k*_*o*_ = *k*_*g*_ = 0 or *π*

**Figure 10:**
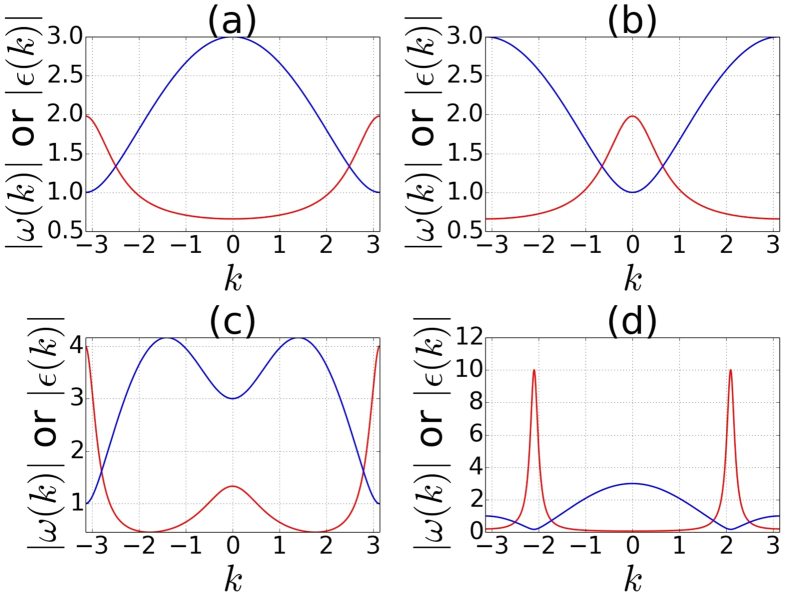
Plots for |*ε*(*k*)| (blue) and |*ω*(*k*)| (red). (**a**) Case 1 with band gap locates at *π* (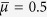 and 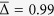). (**b**) Case 1 with band gap locates at 0 (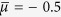 and 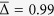). (**c**) The situation belongs to limit 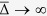 of case 2 (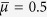 and 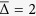). (**d**) The situation belongs to limit 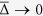 of case 3 (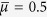 and 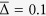).

Consider first the case where both Eqs (22) and (27) do not lead to a valid solution. In this case one has *k*_0_ = 0 or *k*_0_ = *π* and similarly for the *k*_*g*_. Since


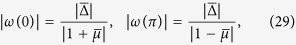


and


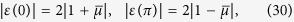


one finds that if 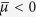 then *k*_0_ = *k*_*g*_ = 0, while if 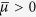 then *k*_0_ = *k*_*g*_ = *π*. Therefore the location of the band gap and the maximum angular velocity are at the same *k*, as shown in Fig. 10(a,b) for 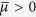 and 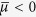 respectively.

• Case 2: Strong paring; 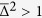


Consider next the case of strong pairing, where 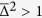. Due to the restriction of 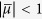 it is straightforward to show that in this case the square root of Eq. (22) is always positive. It is then instructive to consider the two limits of 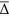: 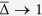 and 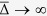.In limit of 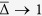, one has


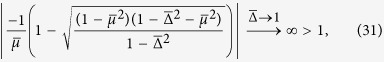


and


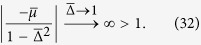


Consequently both Eqs (22) and (27) have no valid solution and one falls back to the scenario of case 1.

In the other limit of 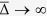, Eq. (22) becomes


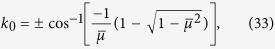


while the Eq. (27) becomes


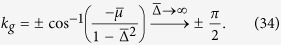


**Figure 11:**
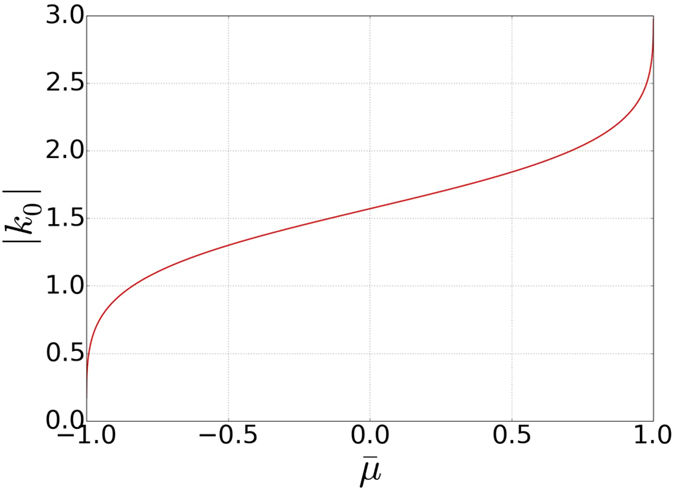
The absolute value of Eq. (33)for the allowed parameter regime 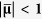. There is always a solution for 0 < |*k*_0_| < *π*.

In Fig. 11 we plot the absolute value of Eq. (33) as a function of 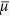 and show that there is always a solution for *k*_0_ within the parameter regime. Due to the 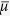 dependence of *k*_0_, in this limit one has *k*_0_ ≠ *k*_*g*_. However, in this limit one also finds


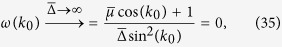



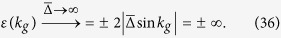


By comparing Eqs (29) and (35), one finds that in this limit Eq. (33) defines a minimum of the angular speed instead of a maximum. Similarly, by comparing Eqs (30) and (36) one finds that the band gap is not located at the momentum defined by Eq. (34). Therefore, we again go back to the scenario of case 1 as shown in Fig. 10(c).

• Case 3: Week paring; 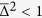


Consider finally the case of weak pairing, where 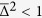. In this case, the term in the square root of Eq. (22) may become negative when 
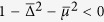
 and gives no valid solution for *k*_0_. However, the same condition also leads to


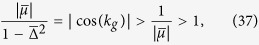


which means there is also no valid solution for *k*_*g*_ according to Eq. (27). Consequently one again falls back to the scenario of case 1.

In order to have a valid solution one requires 
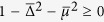
, which means 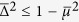. It is then instructive to consider the limits of 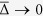 and 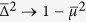. Consider first the limit of 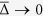 where Eqs (22) and (27) become


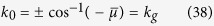


This means that the *k*_0_ and *k*_*g*_ are asymptotically close to each other when the paring goes to zero. On the other hand, in this limit we also have


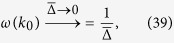



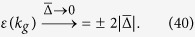


By comparing Eqs (29) and (39), one finds Eq. (39) indeed defines the maximum of the angular velocity. Similarly, Eq. (40) defines the band gap, which can be easily verified by comparing Eqs (30) and (40). Thus, the *k*_0_ and *k*_*g*_ defined in Eqs (22) and (27) are almost the same as shown in Fig. 10(d).

On the other hand, for limit of 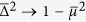, Eqs (22) and (27) become


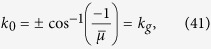


which gives no valid solution for *k*_0_ and *k*_*g*_ due to the restriction of 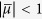 and thus falls back to scenario of case 1.

## Additional Information

**How to cite this article**: Chung, M.-C. *et al*. A Memory of Majorana Modes through Quantum Quench. *Sci. Rep.* **6**, 29172; doi: 10.1038/srep29172 (2016).
